# Variation of Glass Temperature With Pressure in Polypropylene

**DOI:** 10.6028/jres.068A.024

**Published:** 1964-06-01

**Authors:** Elio Passaglia, Gordon M. Martin

## Abstract

By measurement of the specific volume of polypropylene as a function of temperature at various pressures, the variation of glass temperature with pressure, *dT_g_*/*dP*, was determined. Within experimental error the magnitude of this quantity is the same as the value of $T\bar{v}\Delta \alpha /\Delta C_{p}$, where Δ*α* and Δ*C_p_* are the change in coefficient of expansion and specific heat respectively at the glass temperature. This is an indication that thermodynamics can be applied to the glass transition. The value of *dT_g_*/*dP* is the same as Δ*β*/Δ*α*, where Δ*β* is the change in compressibility at *T_g_* calculated from the data, but it is shown that this equality must follow as a consequence of the manner in which the experiments were carried out, quite independently of the application of thermodynamics.

## 1. Introduction

The glass transition as normally observed is almost certainly a consequence of relaxation times associated with molecular motion becoming inordinately long over a small temperature interval as the temperature is lowered to the transition. Nevertheless, the glass transition shows some of the characteristics of an Ehrenfest second-order transition, and indeed a second-order transition associated with the glass transition has been shown to exist on theoretical grounds [1],^1^ and this view has some experimental justification [2, 3].

For a second-order transition, two equations, generally called the Ehrenfest equations, hold:

$$\frac{dT}{dP}=\frac{\Delta \beta }{\Delta \alpha }$$ (1)

$$\frac{dT}{dP}=\frac{T\bar{v}\Delta \alpha }{\Delta C_{p}}$$ (2)

where *T* is the transition temperature, *P* the pressure, $\bar{v}$ the specific volume, and Δ*α*, Δ*β*, and Δ*C_p_* are the changes at the transition in thermal expansion coefficient, compressibility, and the specific heat, respectively.

In spite of the difficulty of applying thermodynamics to the glass [3], it has been shown that these relations would hold for a glass transition [4], provided this is caused by the abrupt change with temperature of some “ordering parameter” *z*, and that the glass can be considered as having a definite “frozen in” value of *z.* In this case, *dT/dP* should be replaced with (∂*T*/∂*P)z.* However, if more than one ordering parameter exists, or more than one type of order changes suddenly at the glass transition, then it is shown that [4]

$$\frac{T\bar{v}\Delta \alpha }{\Delta C_{p}}\leq \frac{\Delta \beta }{\Delta \alpha }.$$ (3)

It is therefore clear that although eqs (1) and (2) of necessity must be obeyed at a second-order transition, the mere fact that they are obeyed does not insure that the transition in question is a second-order transition.

Data of the type necessary to check the applicability of eqs (1) and (2) to the glass transition are relatively scarce for polymers [5, 6]. In particular, measurements of specific heat are lacking. Recently, however, three independent measurements of the specific heat of atactic polypropylene through the glass transition region have been made [2, 7, 8], so that measurements of glass temperature at various pressures for this material would permit checking the applicability of eqs (1) and (2). It is the purpose of this paper to report the result of such measurements.

## 2. Experimental Detail

### 2.1. Apparatus

The apparatus used for measuring volume as a function of temperature at various pressures has been previously described [9]. In this apparatus a glass dilatometer of standard design using mercury as the confining liquid is placed in a chamber in which the pressure may be varied from atmospheric pressure to 1000 kg/cm^2^. The chamber is fitted with windows so that changes of mercury level in the dilatometer tube may be followed with a cathetometer. The whole assembly is placed in a thermostated bath capable of controlling the temperature within ±0.05 °C at any point in the range −30 °C to +150 °C.

### 2.2. Materials

The polypropylene used for this study was provided by the Avisun Corporation, and was the same material as that previously used for calorimetric studies [2]. It had a viscosity average molecular weight of 15,700. The sample was 2 to 3 percent crystalline; hence it contained some isotactic polymer. Evacuation of a specimen in a vacuum desiccator for a week produced a loss of 0.09 percent in weight, so that contamination by solvents was minimal.

### 2.3. Procedure

Measurements of volume were taken between +30 °C and −30 °C. In all cases the temperature was raised to +30 °C, pressure applied, and the temperature lowered in 10, 5, or 2½ deg intervals. At each temperature in and below the transition range a minimum of 1 hr, and often several hours, were allowed for equilibrium to be attained. When the lowest temperature was reached, measurements were repeated by raising the temperature. In most of the experiments the data taken with rising temperature below *T_g_* did not exactly check the data taken with decreasing temperature. This is to be expected when the behavior is relaxational in character.

Data were taken at atmospheric pressure and at pressures of 150, 300, 400, 500, and 700 kg/cm^2^.

The pressure equipment did not permit the attainment of a low enough temperature to give an unambiguous value for *T_g_* at atmospheric pressure. For this measurement, the dilatometer, without the pressure chamber, was placed in another refrigerated bath capable of reaching −40 °C, and measurements were taken down to −38 °C. For this experiment at least 24 hr were allowed for equilibrium for all temperatures below −5 °C, and no difference was observed between the data taken with temperature decreasing or increasing. As will be seen, the glass temperature obtained in this way was somewhat lower than would have been predicted from the other experiments.

## 3. Results

**Figure 1:**
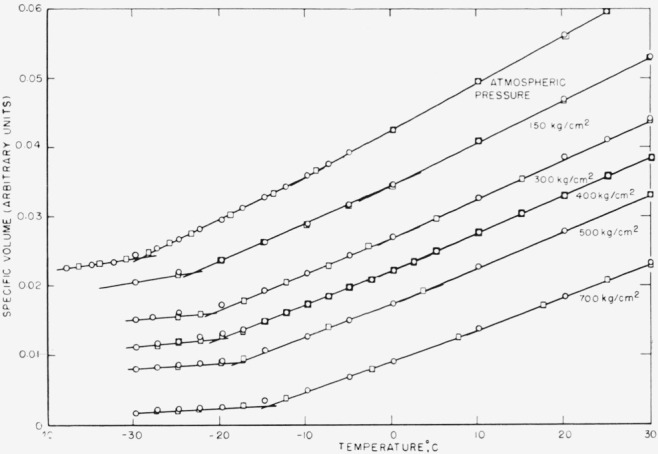
A plot of the specific volume minus the line $\bar{v}=1.150\times 10^{-4}t+1.1075$ as a function of temperature at various pressures Note the evidence for a second break in the curves some 20 to 25 °C above th main break. ○, cooling. □, heating.

From the results of the volume-temperature measurements, the specific volume of the polymer was calculated in the usual way. For ease in plotting the data the arbitrary straight line

$$\bar{v}=1.150\times 10^{-4}t+1.1075,$$

where *t* is the temperature in degrees C, has been subtracted from the observed data and the resulting values are shown in figure 1. It will be apparent that the agreement of the data taken with rising and falling temperature is excellent above the transition temperature.

It will also be apparent that there is a suggestion in each curve of *two* breaks, a small subtle one occurring some 20 deg above the main break. This is much more apparent when plotted on a larger scale. Indeed, it was attempted to fit the curve above the main break by the method of least squares using either two straight lines or a third degree polynomial. Both equations gave the same precision of fit as determined by the residuals, so no judgment about the existence of a second transition could be made on this basis. It cannot be argued that this behavior is due to nonattainment of equilibrium leading to curvature, as always happens near *T_g_*, for the temperature range is too broad, the agreement between the data taken with rising and falling temperatures is excellent, and this type of curvature is limited in the present curves to a region about 5 deg wide near *T_g_.*

Since the present sample does contain some isotactic material and there is evidence that the glass temperature for isotactic polypropylene is higher than that for the atactic [2, 7], it could be assumed that this upper transition is a result of the presence of the isotactic component. This would imply that the atactic polymer is a block copolymer. While such an interpretation of the upper transition is not unreasonable, further discussion would be speculation, and we shall ignore the upper transition, if, indeed, it exists.

The glass temperature was taken as the intersection of the line determined by the lowest temperature data observed with rising temperature, and the line determined by the data for the next 20 or so degrees. If the highest temperature data had been used to determine the $\bar{v}-T$ characteristics of the liquid, all the transition temperatures would be 2 to 3 deg higher, but none of the other conclusions of this paper would be changed.

The glass transition temperatures obtained from these results are shown in table 1. The atmospheric pressure result is somewhat lower than previously reported [2, 7, 8]. Part of the difference may result from the method of analysis, namely, taking the intersection of the lowest straight line with the line defined by the points between −5 and −25 °C, but no doubt part is also due to the very slow rate at which this experiment was conducted.

The table also gives values of $\frac{1}{\bar{v}}\frac{\partial^{v}}{\partial T}$, the coefficient of expansion at *T_g_* for the glass and the liquid, and the difference in this quantity at *T_g_.* To calculate the values of $\partial \bar{v}/\partial T$ above *T_g_* only the highest temperature data were used. The value of Δ*α* at zero pressure determined by a least squares fit of the data for Δ*α* as a function of pressure to a straight line is 4.22×10^−4^ with a computed standard error of 0.12×10^−4^ °C^−1^.

**Figure 2:**
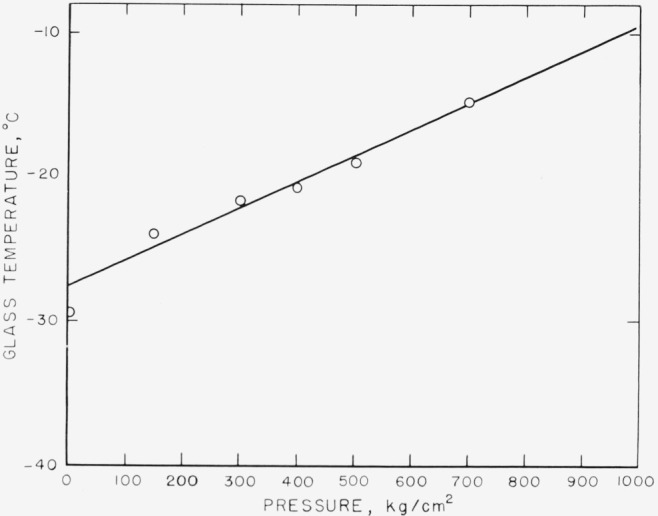
The glass temperature, as determined from figure 1, as a function of gage pressure.

A plot of *T_g_* against the pressure is shown in figure 2. As was noted above, the value of *T_g_* obtained for the experiment at atmospheric pressure is somewhat lower than would have been expected from our other results. This is no doubt due to the slow rate of this experiment. A least squares fit of these data with a straight line gave a value of 0.020 °C/kg cm^−2^ for *dT_g_/dP*, with a computed standard error of 0.002 °C/kg cm^−2^. The value is in accord with measurements on other polymers [5,6].

### 3.1. Compressibility

A determination of the compressibility difference at *T_g_* requires a determination of the compressibility at various temperatures above and below *T_g_* and extrapolation of the results to *T_g_.* Because of the paucity of data below *T_g_*, only one determination, at −30 °C, was made.

**Figure 3:**
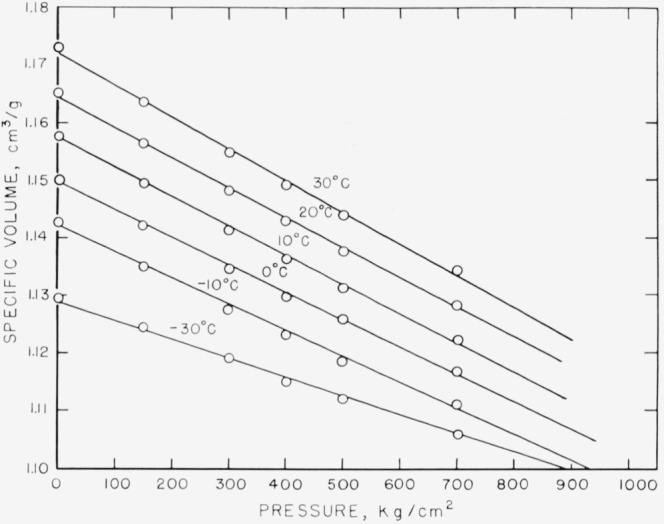
The specific volume at various temperatures as a function of gage pressure The curve at −30 °C is for the material below *T_g_.*

**Figure 4:**
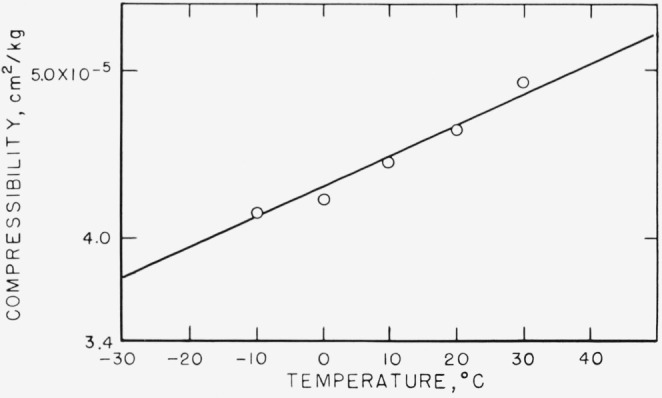
The compressibility, $\frac{-1}{\bar{v}}\frac{\partial \bar{v}}{\partial P}$ at zero pressure, as calculated from figure 3, as a function of temperature. All the points are for material above *T_g_*.

The results for specific volume as a function of pressure at various temperatures are shown in figure 3. The curves require a little comment. The specific volume data could be represented by slightly curved lines curving in the direction to be expected [10]. For the purposes of this paper, the curves were approximated by straight lines, which is an adequate approximation to the volume-pressure relation in this pressure range. The line at −10 °C was obtained by extrapolating the volume-temperature curves from higher temperatures to this temperature. The compressibilities, which were calculated from these lines by dividing the slope by the specific volume at zero pressure at the temperature in question, are shown in figure 4, and listed in table 2. The value of the compressibility of the liquid extrapolated to −30 °C is also given. The value of Δ*β* at this temperature is 0.87×10^−5^ cm^−2^/kg, with limits of error estimated subjectively not to exceed ±0.04×10^−5^ cm^2^/kg.

### 3.2. Comparison of Results

Table 3 gives a comparison of the value of *dT_g_*/*dP* with the values of Δ*β*/Δ*α* and $T\bar{v}\Delta \alpha /\Delta C_{p}$. The latter was calculated using the specific heat results of Passaglia and Kevorkian [2]; Dainton, Evans, Hoare, and Melia [7]; Wilkinson and Dole [8]. The agreement among the three quantities is seen to be quite satisfactory.

## 4. Discussion

The agreement between the values of *dT_g_*/*dP* and $T\bar{v}\Delta \alpha /\Delta C_{p}$ is in accord with the previous results of O’Reilly [5] on polyvinyl acetate, and we concur with his conclusion that thermodynamics may be applied to the glass transition.

The agreement between $T\bar{v}\Delta \alpha /\Delta C_{p}$ and Δ*β/*Δ*α* requires more comment. O’Reilly did not observe agreement between these two quantities: they found $\Delta \beta /\Delta \alpha >T\bar{v}\Delta \alpha /\Delta C_{p}$, which would imply more than one “ordering parameter” at the glass transition. In our own case the agreement between Δ*β/*Δ*α* and $T\bar{v}\Delta \alpha /\Delta C_{p}$ would imply either a single ordering parameter or indeed a true second-order transition.

However, the agreement between Δ*β/*Δ*α* and *dT_g_*/*dP* is not a consequence of thermodynamics, but a consequence of the manner in which the experiments were carried out and the results analyzed. For consider the idealized case of figure 1: below the glass-transition temperature, any one of the normalized volume-temperature curves can be represented in the region of interest by

$$\frac{\bar{v}}{{\bar{v}}_{g}}=\gamma_{g}+\alpha_{g}T$$ (4)

where ${\bar{v}}_{g}$ is the specific volume at *T_g_, α_g_* is the coefficient of expansion, and *γ_g_* is a constant for a particular curve. These quantities are, of course, functions of the pressure. Above *T_g_* the analogous equation is

$$\bar{v}/{\bar{v}}_{g}=\gamma_{l}+\alpha_{l}T.$$ (5)

Clearly *T_g_* is given by the intersection of these two lines, or

$$T_{g}=\frac{\gamma_{l}-\gamma_{g}}{\alpha_{g}-\alpha_{l}}=\frac{-\Delta \gamma }{\Delta \alpha }.$$ (6)

Consequently, we obtain

$$dT_{g}/dT=-\frac{1}{\Delta \alpha }\frac{d\Delta \gamma }{dP}+\frac{\Delta \gamma }{\Delta \alpha^{2}}\frac{d\Delta \alpha }{dP}$$ (7)

which relates the change with pressure of *T_g_* with the pressure variations of the constants of the $\bar{v}-T$ curves above and below *T_g_.* Now consider how Δ*β* is determined from data of this type. By definition, we have,

$$\beta =-\frac{1}{\bar{v}}\frac{\partial \bar{v}}{\partial P}.$$

Hence we have, using eqs (4) and (5)

$$\beta_{g}=-\frac{{\bar{v}}_{g}}{\bar{v}}\left(\frac{d\gamma_{g}}{dP}+T\frac{d\alpha_{g}}{dP}\right)-\frac{1}{\bar{v}}(\gamma_{g}+\alpha_{g}T)\frac{d{\bar{v}}_{g}}{dP}$$

and

$$\beta_{l}=-\frac{{\bar{v}}_{g}}{\bar{v}}\left(\frac{d\gamma_{l}}{dP}+T\frac{d\alpha_{l}}{dP}\right)-\frac{1}{\bar{v}}(\gamma_{l}+\alpha_{l}T)\frac{d{\bar{v}}_{g}}{dP}.$$ (8)

At *T=T_g_*, noting that *γ_l_ +α_l_T_g_=γ_g_+α_g_T_g_*=1, we have

$$\Delta \beta =\beta_{l}-\beta_{g}=\frac{-d\Delta \gamma }{dP}-T_{g}\frac{d\Delta \alpha }{dP}.$$ (9)

Using the value of *T_g_* from eq (6), we obtain finally

$$\frac{\Delta \beta }{\Delta \alpha }=-\frac{1}{\Delta \alpha }\frac{d\Delta \gamma }{dP}+\frac{\Delta \gamma }{\Delta \alpha^{2}}\frac{d\Delta \alpha }{dP}$$

which is identical to eq (7). Hence as long as experiments are performed in this way and as long as the volume-temperature curves can be approximated by straight lines over the region of interest, Δ*β*/Δ*α must* be equal to *dT_g_*/*dP.*

To say this another way, these measurements of $\bar{v}$ as a function of *T* and *P* define a surface in $\bar{v}$, *T*, *P* space. So long as we operate only on this surface, then eq (1) follows as a geometric consequence, even though the surface may not represent a thermodynamic equilibrium surface. Only if we were to do another experiment (e.g., measure $\bar{v}$ as a function of *P* at various temperatures) to map out the same surface could we make any statement about the applicability of thermodynamics.

## 5. Conclusions

By measurement of specific volume as a function of temperatures at various pressures, *dT_g_*/*dP* and Δ*β*/Δ*α* were calculated. These quantities agreed within experimental error, and it is shown that they must. The agreement of these quantities with $T\bar{v}\Delta \alpha /\Delta C_{p}$ indicates that thermodynamics may be applied to the glass transition.

## Figures and Tables

**Table 1:** Glass transition temperatures and coefficients of expansion at *T_g_* for the glass and liquid

*P*	*T_g_*	*α_g_*	*α_l_*	Δ*α*

*kg/cm*^2^	°*C*	°*C*^−1^	°*C*^−1^	°*C*^−1^
0	−29.5	2.39×10^−4^	6.80×10^−4^	4.41×10^−^
150	−24.0	2.64	6.47	3.83
300	−21.7	2.14	6.09	3.95
400	−20.7	2.06	5.89	3.83
500	−19.0	1.29	5.68	3.79
700	−14.7	1.62	5.24	3.62

**Table 2:** Compressibility at various temperatures for the glass and liquid

*T*	*β_l_*	*β_g_*

*°C*	*cm^2^/kg*	*cm*^2^/*kg*
30	4.93×10^−5^	………
20	4.62	………
10	4.46	………
0	4.23	………
−10	4.16	………
−30	13.77	2.90×10^−5^

1 Extrapolated value.

**Table 3:** Values of terms in the Ehrenfest equations

*dT_g_/dP*	Δ*β*Δ*α*	$T\bar{v}\Delta \alpha /\Delta C_{p}$

*°C/kg cm*^−2^	°*C/kg cm*^−2^	*°C/kg cm*^−2^
0.020 ±0.004*	0.021 ±0.002†	0.024 ±0.001‡

* Twice the computed standard error.

† Subjectively estimated maximum error.

‡ Computed from the error in Δ*α* alone; *T*, $\bar{v}$, Δ*C_p_* considered to be error free.

## Footnotes

1 Figures in brackets indicate the literature reference at the end of this paper.
